# Practical Guidelines for the Use of Indocyanine Green in Different Branches of Pediatric Surgery: A Polish Nationwide Multi‐Center Retrospective Cohort Study

**DOI:** 10.1002/hsr2.70586

**Published:** 2025-03-23

**Authors:** Patrycja Sosnowska‐Sienkiewicz, Grzegorz Kowalewski, Hanna Garnier, Anna Wojtylko, Maciej Murawski, Marta Szczygieł, Magdalena Al‐Ameri, Piotr Czauderna, Jan Godzinski, Piotr Kalicinski, Przemysław Mankowski

**Affiliations:** ^1^ Department of Pediatric Surgery, Traumatology and Urology Poznan University of Medical Sciences Poznań Poland; ^2^ Department of Pediatric Surgery and Organ Transplantation The Children's Memorial Health Institute Warsaw Poland; ^3^ Department of Surgery and Urology for Children and Adolescents Medical University of Gdansk Gdansk Poland; ^4^ Department of Pediatric Surgery Marciniak Hospital Wroclaw Poland

**Keywords:** children, fluorescence, ICG, indocyanine green, intraoperative assessment, pediatric surgery, technology

## Abstract

**Background and Aim:**

Pediatric surgery requires high precision and safety due to children's unique anatomy and physiology. Innovations that enhance surgical precision, reduce operative time, and improve postoperative outcomes are invaluable. Indocyanine green (ICG), a near‐infrared fluorescent dye, has received significant attention for its potential to address these needs. The study aimed to describe applications of ICH in pediatric surgery in Poland, provide evidence to support integration of ICG into pediatric surgical practice and evaluate the safety and feasibility of ICG.

**Methods:**

Data were collected from the medical records of 136 patients undergoing surgical treatment in four leading pediatric surgical clinical centers in Poland. There are four main groups of surgical procedures: oncological, gastrointestinal, urological and lymphatic. Calculations were performed using Statistica 13 by TIBCO and PQStat v. 1.8.6.120 by PQStat Software. Additionally, descriptive statistics were performed.

**Results:**

The efficacy and safety of ICG were confirmed in the entire study group. No complications were reported with the use of the indocyanine green. Exhaustive descriptive statistics presented key information on the use of ICG for each of the studied groups.

**Conclusions:**

The study validates the efficacy and safety of ICG fluorescence imaging in pediatric surgery. By enhancing surgical precision and improving postoperative outcomes, ICG represents a significant advancement in pediatric surgical care. The establishment of standardized protocols and the emphasis on specialized training will be key to maximizing the benefits of ICG.

## 1 Introduction

Pediatric surgery requires a high level of precision and safety due to the unique anatomy and physiology of children. Innovations that enhance surgical precision, reduce operative time, and improve postoperative outcomes are invaluable [1, 2]. Fluorescence guided surgery (FGS) is based on the principle of Stokes shift and refers to the energy difference between the absorption and emission spectra of fluorescent contrast agents. Using an external light source, the fluorescent agent is excited at a specific wavelength that increases the energy level of the fluorescent molecule for ~10–8 s [2, 3]. The difference between the excited and emitted light is recorded by the sensors and displayed on the monitor in real time. Indocyanine green (ICG) is one of the most commonly used near‐infrared fluorophores in FGS [2, 3]. It is a water‐soluble dye developed in 1955 for use in the motion picture industry. Irwin J. Fox and Earl H. Wood of the Mayo Clinic first described its potential use in medicine to assess cardiac output, leading to its approval in 1956 for use in humans to assess liver and heart function. Known for its rapid vascular clearance, minimal toxicity, and high tissue penetration, ICG offers a versatile and powerful tool for a range of surgical applications [2, 3]. ICG binds preferentially to albumin and distributes uniformly throughout the intravascular compartments, allowing assessment of vascular flow and anatomy. It has a good safety profile, except in rare cases of allergy or in patients with thyroid disorders due to the 5% iodine salt content in the preparation. It can also be used in newborns. One of the primary applications of ICG in pediatric surgery is intraoperative imaging. ICG fluorescence imaging allows real‐time visualization of blood vessels, lymphatic vessels and tissue perfusion. ICG is excited by near infrared (NIR) light at a wavelength of approximately 760–790 nm and is visualized at a peak emission wavelength of approximately 830 nm [1]. The depth of detection varies depending on the type of tissue and the depth of penetration of the NIR light, typically between 5 and 10 mm [1]. The hepatic clearance and biliary excretion properties of ICG also facilitate the assessment of liver function and the visualization of the biliary tree during various procedures, that is, during cholecystectomy, biliary cyst resection, or the Kasai procedure. In gastrointestinal surgery, ICG also can help assess intestinal blood flow. The short half‐life of 2–4 min allows for multiple injections [1, 4, 5].

The role of ICG is particularly important in oncologic surgery. In some lesions, it can aid in the delineation of tumor margins and the identification of lymph nodes. It may help determine the location of lung metastases. By providing real‐time visualization, ICG holds the promise of more precise surgical excisions [1, 4, 5, 6, 7].

In addition, ICG has also shown promise in reconstructive surgery. For example, ICG can help identify and preserve vital structures in complex congenital anomalies such as esophageal atresia, minimizing the risk of postoperative complications [4]. Similarly, in urological procedures, ICG facilitates the identification of ureters and renal vasculature. In this situation, a catheter is inserted into the ureter under the guidance of a cystoscope, and ICG is administered through the catheter [8, 9].

This study not only highlights the diverse applications of ICG, but also examines the practical aspects of its use, such as dosage and administration techniques. Another new experience in the use of ICG was also presented. By compiling comprehensive data from multiple centers, the study aims to establish standardized protocols and best practices for the use of ICG in pediatric surgery. It also addresses potential challenges and limitations, such as the non‐specificity of the preparation, variability of fluorescence equipment, still limited experience with its use, lack of standardized methods of ICG application, and the need for specialized training [3, 6].

This publication details the results of a Polish nationwide, multicenter cohort study that was carefully designed to evaluate the applicability, efficacy, and safety of ICG in various areas of pediatric surgery. This comprehensive study draws on data from numerous healthcare institutions across Poland and aims to provide a holistic view of the applications of ICG in the pediatric surgical landscape.

Through this nationwide cohort study, we aimed to describe applications of ICG in pediatric surgery in Poland, provide evidence to support integration of ICG into pediatric surgical practice and evaluate the safety and feasibility of ICG. This study will not only contribute to the existing body of knowledge but also pave the way for future innovations and improvements in the field of pediatric surgery.

## 2 Materials and Methods

Data were collected from the medical records of patients undergoing surgical treatment in four leading pediatric surgical clinical centers in Poland with equipment for intraoperative visualization of indocyanine green. The study included 136 patients operated with the use of ICG imaging in the following centers in the period 2020–2024: Department of Pediatric Surgery, Traumatology and Urology in Poznan, Department of Pediatric Surgery and Organ Transplantation in Warsaw, Department of Surgery and Urology for Children and Adolescents in Gdansk, Department of Pediatric Surgery in Wroclaw (Table 1).

**Table 1:** Characteristics of a group of patients operated with using ICG.

Parameter	*N*	%
Number of patients	136	100
Sex		
Girls	65	48
Boys	71	52
Age		
Range (months)	35–174	—
Mean (months)	100	—
Median (months)	76	—
Group of surgery		
Oncological	82	60
Gastrointestinal	34	25
Urological	11	8
Lymphadenectomy and sentinel node biopsy	9	7
Surgical access		
Laparoscopy	60	44
Laparotomy	22	16
Thoracoscopy	20	15
Thoracotomy	10	7
Other (e.g., sentinel node biopsy)	24	18
Confirmation of the initial surgical assessment through histopathology		
Yes	114	84
No	22	16

The analyzed surgical procedures utilized the Stryker 1688 advanced imaging modalities 4K and SPY‐PHI platform, which offer both standard white light imaging and NIR fluorescence imaging capabilities. The ICG dye (Verdye, Pulsion Medical Systems, Munich, Germany), available in vials (25 mg), was adopted in all procedures.

One hundred thirty‐six patients underwent surgical treatment during which indocyanine green was used. These procedures were performed using both open‐access and minimally invasive techniques and included gastrointestinal, oncological, urological surgeries and operations including lymphadenectomy and sentinel node biopsy (Table 2).

**Table 2:** Final diagnosis after histopathological examination in each operating group.

Diagnosis	*N* (136)	%
Oncological surgery group		
Adrenal gland tumor		3
Neuroblastoma	1	
Pheochromocytoma	3	
Renal tumor		11
Nephroblastoma	13	
Renal cell carcinoma	1	
Pediatric cystic nephroma	1	
Hepatic tumor		26
Hepatoblastoma	21	
Hepatocarcinoma	6	
Rhabdomyosarcoma	1	
Hepatic mesenchymal hamartoma	1	
Liver cyst	1	
Desmoplastic small‐round‐cell tumor	1	
Undifferentiated sarcoma	1	
Vascular tumor of the liver	3	
Ovarian tumor		5
Teratoma mature	6	
Teratoma immature	1	
Pancreatic tumor		2
Pancreatic neuroendocrine tumor	1	
Solid pseudopapillary neoplasm	1	
Persistent hyperinsulinemic hypoglycemia of infancy	1	
Thyroid tumor		0.7
Follicular thyroid cancer	1	
Lung lesions		2
Osteosarcoma metastasis	1	
Ewing sarcoma metastasis	1	
Pulmonary histiocytosis	1	
Other	10	7
Gastrointestinal surgery group		
Cholecystolithiasis	24	18
Choledochal cyst	5	4
Esophageal atresia	1	0.7
Chemical burn of the esophagus	1	0.7
Hirschsprung disease	1	0.7
Ulcerative colitis	1	0.7
Cholestasis	1	0.5
Abdominal lymphadenopathy	1	0.5
Urological surgery		
Varicocele	8	6
Renal atresia	3	2
Lymphadenectomy and sentinel node biopsy		
Malignant melanoma	6	4
Renal tumor, lymphadenectomy		2
Nephroblastoma	3	
Reactive lymph node	2	1
Other	1	0.7

We divided our cohort into groups based on the type of surgery performed.

The group of oncological surgeries included adrenal tumor resection, liver tumor resection, lung lesion surgery, pancreatic tumor resection, nephrectomy (for oncological indications), resection of abdominal metastatic lesions, lymphadenectomy, sentinel node biopsy in melanoma, ovarian surgery, thyroid surgery (Tables 3, 4, 5). The group of gastrointestinal operations included biliary surgery, esophageal reconstruction with small intestine, intestinal resection and anastomosis (Table 6). The urological procedures included varicocelectomy and nephrectomy for non‐oncological indications (Table 7).

**Table 3:** The way ICG was used during oncological surgeries.

Purpose of ICG administration	Dose	Dilution (mg ICG/1 ml sterile aqua)	Route of administration	Administration time	Effect of ICG administration achieved
Adrenal gland tumor surgery					
Pheochromocytoma (Figure 1)	5 mg (1 mL)	5	To a peripheral vein	Intraoperatively	Hyper‐/hypo‐fluorescence of the tumor in the gland
Renal tumor surgery	0.5 mg/kg body weight	2.5	To a peripheral vein	Intraoperatively	Fluorescence of healthy kidney part (without tumor)
	1.5 mg/kg body weight	2.5	To a peripheral vein	24 h before the surgery	Fluorescence of the tumor
Nephroblastoma metastasis	1.25 mg/kg body weight	5	To a peripheral vein	24 h before the surgery	Fluorescence of the metastasis
Hepatic tumor surgery					
Hepatoblastoma/Hepatocarcionoma (Figure 2)	0.5 mg/kg body weight	5	To a peripheral vein	48–72 h before the surgery	Fluorescence of the tumor
Rhabdomyosarcoma	1.25 mg/kg body weight	5	To a peripheral vein	48 h before the surgery	Fluorescence of the tumor
Vascular tumor of the liver (Figure 3)	0.5 mg/kg body weight	5	To a peripheral vein	24 h before the surgery and intraoperatively	Fluorescence of the healthy part of the liver; ICG quickly flushed out of tumor
Ovarian tumor surgery (Figure 4)	0.5 mg/kg body weight	2.5	To a peripheral vein	Intraoperatively	Fluorescence of healthy ovary part
Pancreatic tumor surgery					
Insulinoma	5 mg	5	To a peripheral vein	Intraoperatively	Hiperfluorescence of the tumor
Thyroid tumor surgery	5 mg	2.5	To a peripheral vein	Intraoperatively	Fluorescence of the parathyroid gland
Lung lesions surgery					
Hepatoblastoma/hepatocarcinoma/osteosarcoma metastasis (Figure 5)	0.5 mg/kg body weight	5	To a peripheral vein	24 h before the surgery	Fluorescence of the lesions
Pulmonary histiocytosis	1.25 mg/kg body weight	5	To a peripheral vein	24 h before the surgery	Fluorescence of the lesions

**Table 4:** The false positive and false negative results in the oncological surgery group.

Diagnosis	False positive result	False negative result
Hepatoblastoma	1. Visualization of fluorescent foci in the diaphragm during surgery. Histopathology showed no evidence of malignancy.	1. Resection of pulmonary lesion. No fluorescence of the lesion during thoracoscopy, conversion to thoracotomy – fluorescence of the lesion after manual lung compression.
	2. Widening of the resection margin due to ICG fluorescence in the liver. No malignant features on histopathology.	2. Resection of pulmonary lesion. No fluorescence of the lesion during thoracoscopy, conversion to thoracotomy – fluorescence of the lesion after manual lung compression.
	3. Widening of the resection margin due to ICG fluorescence in the liver. No malignant features on histopathology.	—
	4. Suspicion of pulmonary metastases. Fluorescence of lesions during thoracoscopy. No malignant features on histopathology.	—
Hepatocarcinoma	5. Suspicion of pulmonary metastases. Fluorescence of lesions during thoracoscopy, in histopathology atelectasis, pulmonary congestion.	3. Resection of the liver tumor. Illumination of the tumor perimeter only.
	6. Suspicion of pulmonary metastases. Fluorescence of lesions during thoracoscopy, in histopathology atelectasis, pulmonary congestion.	—
Solid pseudopapillary neoplasm	7. Uniform fluorescence throughout the pancreas. No significant effect of dose.	—
Nephroblastoma	8. Suspicion of pulmonary metastases. Fluorescence of lesions during thoracoscopy Histopathology showed no evidence of malignancy.	4. Resection of a tumor located centrally in the kidney‐ no intraoperative fluorescence after ICG administration.
Persistent hyperinsulinemic hypoglycemia of infancy	9. Uniform fluorescence throughout the pancreas. No significant effect of dose.	—
Ewing sarcoma	—	5. Resection of pulmonary lesion. No fluorescence of the lesion during thoracoscopy, conversion to thoracotomy‐ 1 lesion.

**Table 5:** The way ICG was used during lymphadenectomy and sentinel node biopsy.

Purpose of ICG administration	Dose	Dilution (mg ICG/1 mL sterile aqua)	Route of administration	Administration time	Effect of ICG administration achieved
Lymphadenectomy in nephroblastoma (Figure 9)	1 mg	1	Injection into the healthy part of the kidney (four sites)	Intraoperatively	Fluorescence of lymphatic vessels and nodes
Sentinel node biopsy (melanoma) (Figure 10)	1 mg or 2.5 mg	1 or 2.5	Intradermally, around the lesion	Intraoperatively	Fluorescence of lymphatic vessels, fluorescence sentinel node

**Table 6:** The way ICG was used during gastrointestinal procedures.

Purpose of ICG administration	Dose	Dilution (mg ICG/1 mL sterile aqua)	Route of administration	Administration time	Effect of ICG administration achieved
Resection of the gallbladder and choledochal cyst (Figure 6)	0.5 mg/kg body weight	2.5 or 5	To a peripheral vein	24 h before the surgery	Fluorescence of the bile ducts
Assessment of perfusion during					
Esophageal reconstruction with small intestine	5 mg	5	To a peripheral vein	Intraoperatively	Selection of an intestinal insert. Confirmation of adequate blood supply during the creation of the intestinal conduit.
During intestinal anastomosis (Figure 7)	5 mg	5	To a peripheral vein	Intraoperatively	Fluorescence confirmation of normal bowel vascular perfusion.

**Table 7:** The way ICG was used during urological surgeries.

Purpose of ICG administration	Dose	Dilution (mg ICG/1 ml sterile aqua)	Route of administration	Administration time	Effect of ICG administration achieved
Varicocele surgery (Figure 8)	6.25 mg or 5 mg	5	To the testis	Intraoperatively	Fluorescence of lymphatic vessels
Resection of the atrophic kidney	0.5 mg/kg body weight	2.5	To a peripheral vein	Intraoperatively	Fluorescence of the atrophic kidney

The following parameters were assessed in the study: age, gender, primary diagnosis/indications for surgery, surgical technique, planned procedure, implementation of the planned aim, method of ICG administration (dose, time, and route of administration), the usefulness of ICG in visualizing the lesion, histopathological confirmation of the collected material.

This descriptive paper aims to present phenomena, data, or qualitative analyses of ICG use in pediatric patients. No statistical methods or comparative analyses were applied, as this aligns with the methodological assumptions and scope of the study. Any conclusions drawn in the article should be regarded as descriptive findings rather than results based on quantitative hypothesis verification.

Adverse effects of ICG administration were defined as any undesired reactions or complications observed following the intravenous injection of ICG. These included but were not limited to allergic reactions (e.g., rash, itching, or anaphylaxis), cardiovascular effects (e.g., hypotension or tachycardia), and any signs of toxicity such as nausea or vomiting. The presence of adverse effects was systematically assessed and recorded during and after the procedure.

The study was approved by the Bioethics Committee of the Medical University of Poznan (Resolution No. KB‐699/23 of September 20, 2023). All procedures performed in studies involving human participants were in accordance with the ethical standards of the institutional and/or national research committee and with the 1964 Helsinki declaration and its later amendments or comparable ethical standards.

## 3 Results

The efficacy and safety of ICG were confirmed in the entire study group. Complication rates were assessed in every patient. There were no complications related to ICG administration in our cohort.

Exhaustive descriptive statistics were summarized for each of the studied groups.

## 4 Study Group Characteristics

Our experience has been summarized in tables to facilitate the use of green indocyanine in daily surgical practice. Each table provides practical tips on how to use indocyanine green depending on the goal the operator is trying to achieve.

## 5 Oncological Treatment Group

**Figure 1:**
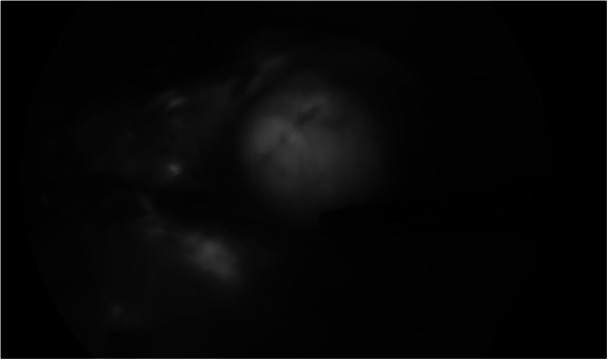
Hyperfluorescence of pheochromocytoma of the adrenal gland. Hypofluorescent focci in the background also including pheochromocytoma. The image was captured intraoperatively in monochromatic NIR/ICG mode.

**Figure 2:**
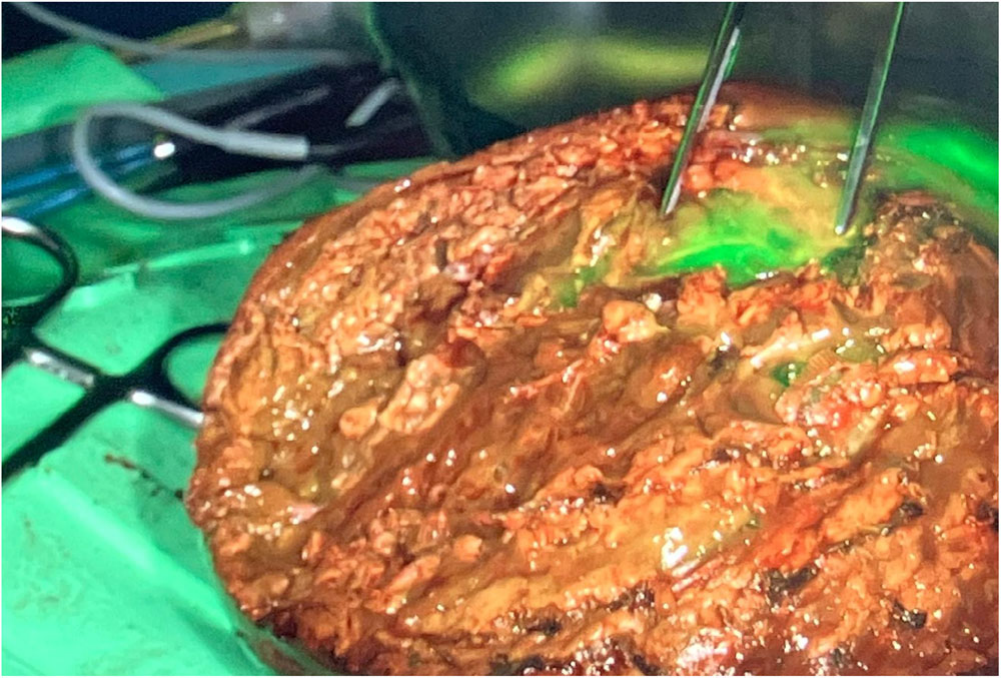
Resection of a hepatoblastoma. ICG fluorescence suggested possible tumor involvement on the resection plane; histopathological examination confirmed clear surgical margins.

**Figure 3:**
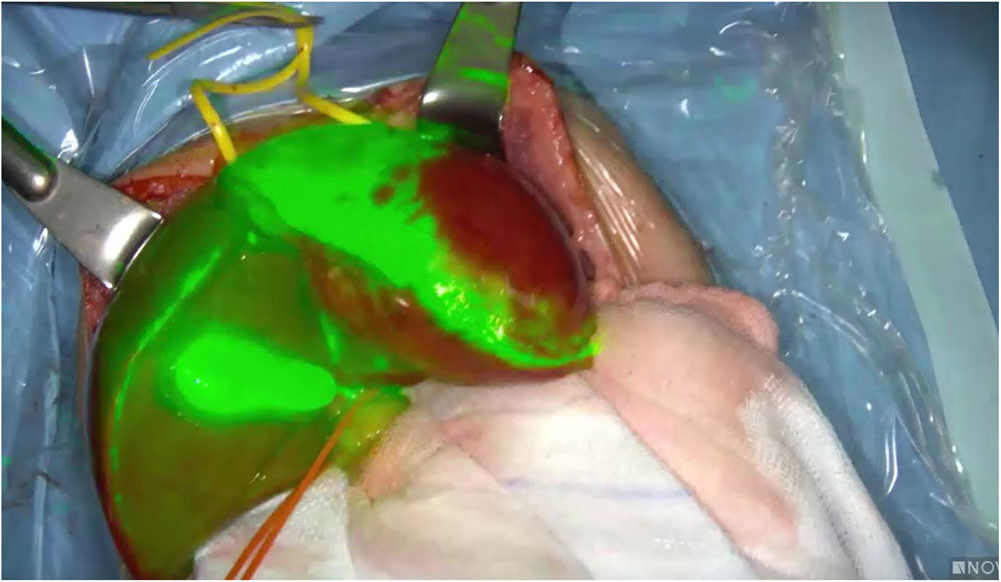
Fluorescence image during resection of a vascular liver lesion. ICG was washed out of the lesion. Fluorescence of the healthy part of the liver was observed.

**Figure 4:**
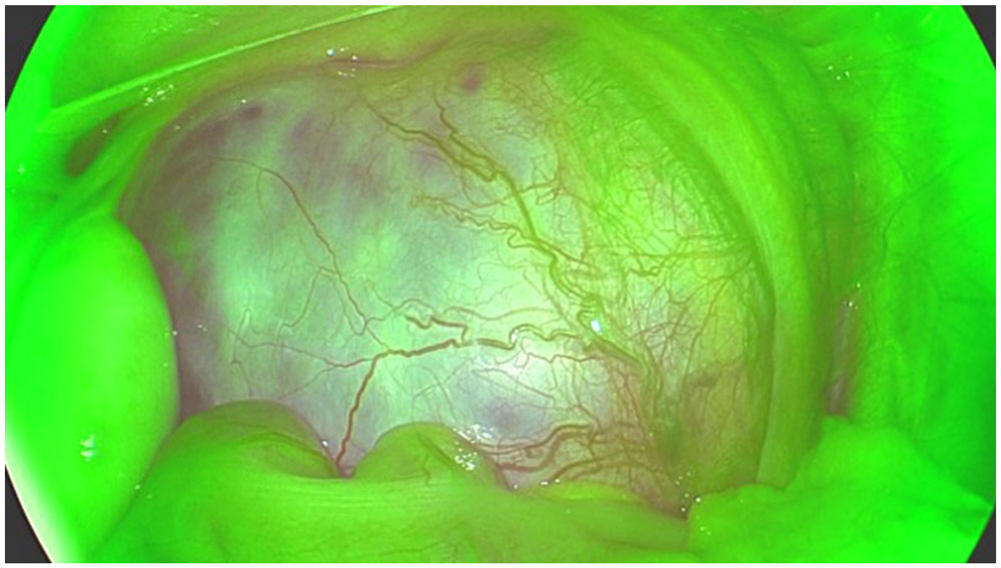
Clearer fluorescence of a healthy ovarian tissue compared to tumor lesion.

**Figure 5:**
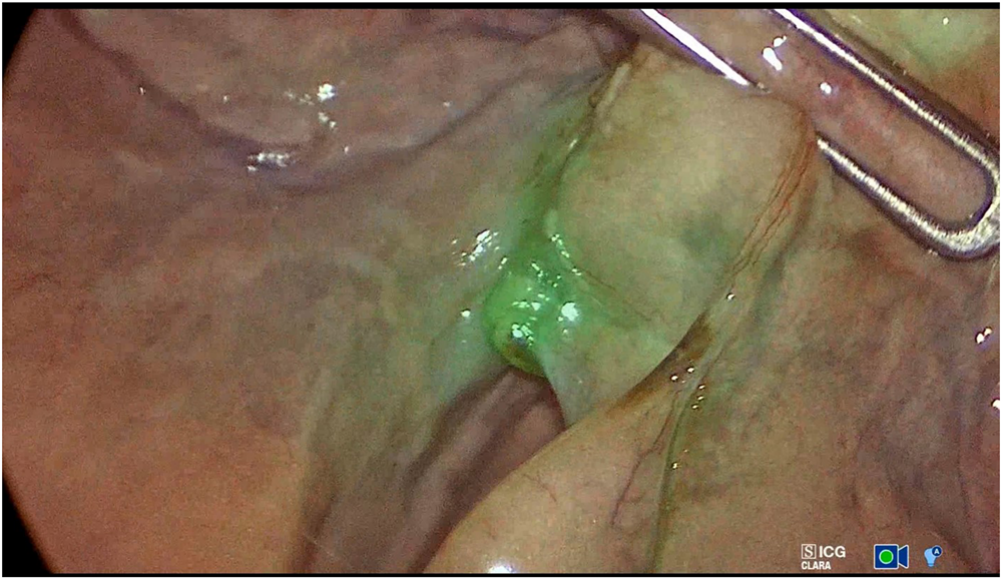
Fluorescence of lung lesions in metastatic osteosarcoma.

All of the techniques described above showed efficacy in the study group.

When more than one way of ICG administration was described, each of them had the same effectiveness of intraoperative visualization (e.g., for Wilms' tumor, both preoperative and intraoperative application of ICG showed comparable results [as judged by the operating surgeon], as did the administration of a dilution of 2.5 or 5 to visualise the gallbladder and bile ducts).

Situations where, according to our observations, we did not notice any kind of visualization effect (24 patients), are described in Table 8.

**Table 8:** Situations where ICG was administered and no visualization was obtained.

Purpose of ICG administration	Final diagnosis	Dose	Dilution (mg ICG/1 mL sterile aqua)	Route of administration	Administration time	Number of observed patients
Adrenal gland resection	Neuroblastoma	5 mg	5	To a peripheral vein	20 h before surgery	1
Adrenal gland resection	Pheochromocytoma	5 mg	5	To a peripheral vein	Intraoperatively	1
Resection of the pancreatic tumor	Solid pseudopapillary neoplasm	0.5 mg/kg body weight	5	To a peripheral vein	24 h before surgery and intraoperatively	1
Resection of the pancreatic lesions	Persistent hyperinsulinemic hypoglycemia of infancy	1 mg/kg body weight	5	To a peripheral vein	Intraoperatively	1
Resection of the lung metastasis	Ewing sarcoma	1.5 mg/kg body weight	5	To a peripheral vein	Intraoperatively	2
Resection of the lung metastasis	Nephroblastoma	1.5 mg/kg body weight	5	To a peripheral vein	24 h before surgery	3
Resection of the abdominal metastatic lymph nodes	Nephroblastoma	1 mg/kg body weight	5	To a peripheral vein	24 h before surgery	1
Resection of the abdominal metastatic lymph nodes	Nephroblastoma	5 mg	1	To perirenal fat	Intraoperatively	2
Resection of the abdominal metastatic lymph nodes	Renal cell carcinoma	5 mg	1	To perirenal fat	Intraoperatively	1
Resection of the liver metastasis	Osteosarcoma	3 mg/kg body weight	5	To a peripheral vein	24 h before surgery	1
Resection of the liver tumor	Desmoplastic small round cell tumor	75 mg	5	To a peripheral vein	24 h before surgery	1
Resection of the liver tumor	Cavernous hemangioma	10 mg	5	To a peripheral vein	72 h before surgery	1
Resection of the liver tumor	Liver cyst	10 mg	5	To a peripheral vein	72 h before surgery	1
Lymphadenectomy	Pediatric cystic nephroma	5 mg	1	To perirenal fat	Intraoperatively	1
Nephron‐sparing surgery	Nephroblastoma	1.5 mg/kg body weight	2.5	To a peripheral vein	24 h before surgery	1

The oncologic surgery group was the only group with false positives (9 patients) and false negatives (5 patients) results. The listed situations were collected in Table 4 and summarized by diagnosis. However, the reasons for the described observations were not found.

## 6 Lymphadenectomy and Sentinel Node Biopsy

## 7 Gastrointestinal Treatment Group

**Figure 6:**
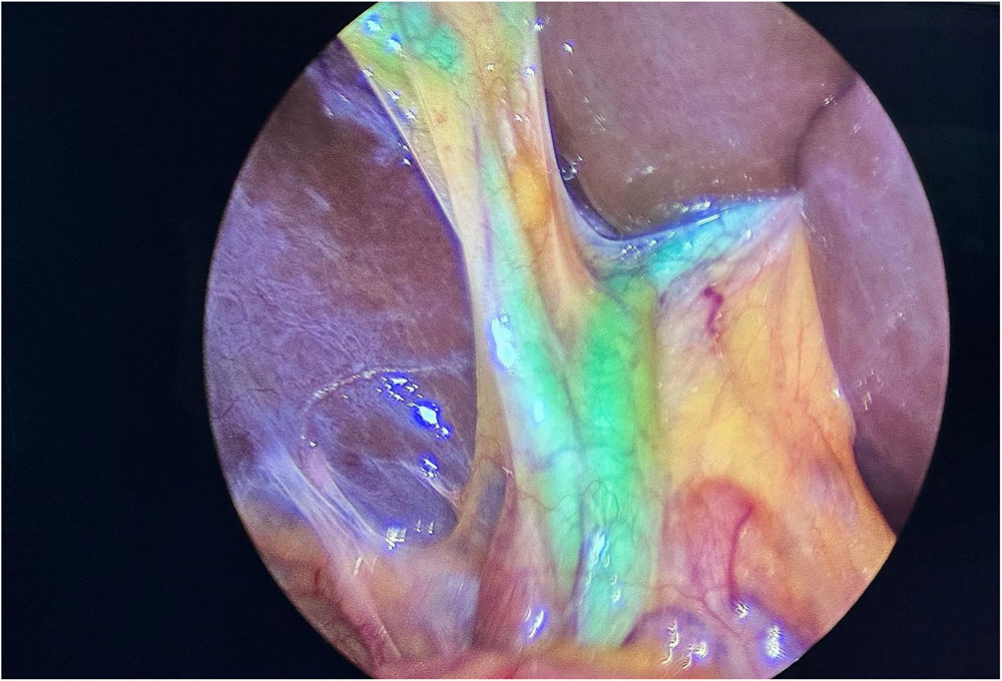
Visualization of the bile ducts during cholecystectomy surgery.

**Figure 7:**
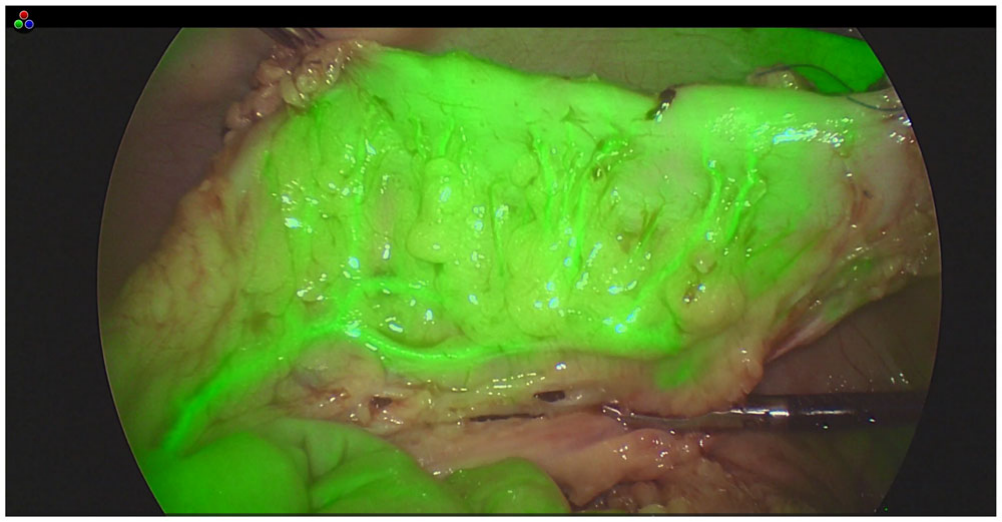
Assessment of intestinal perfusion during laparoscopic surgery for Hirschsprung's disease before Duhamel's anastomosis after closure of a vascular arcade before final colorectal anastomosis.

## 8 Urological Treatment Group

**Figure 8:**
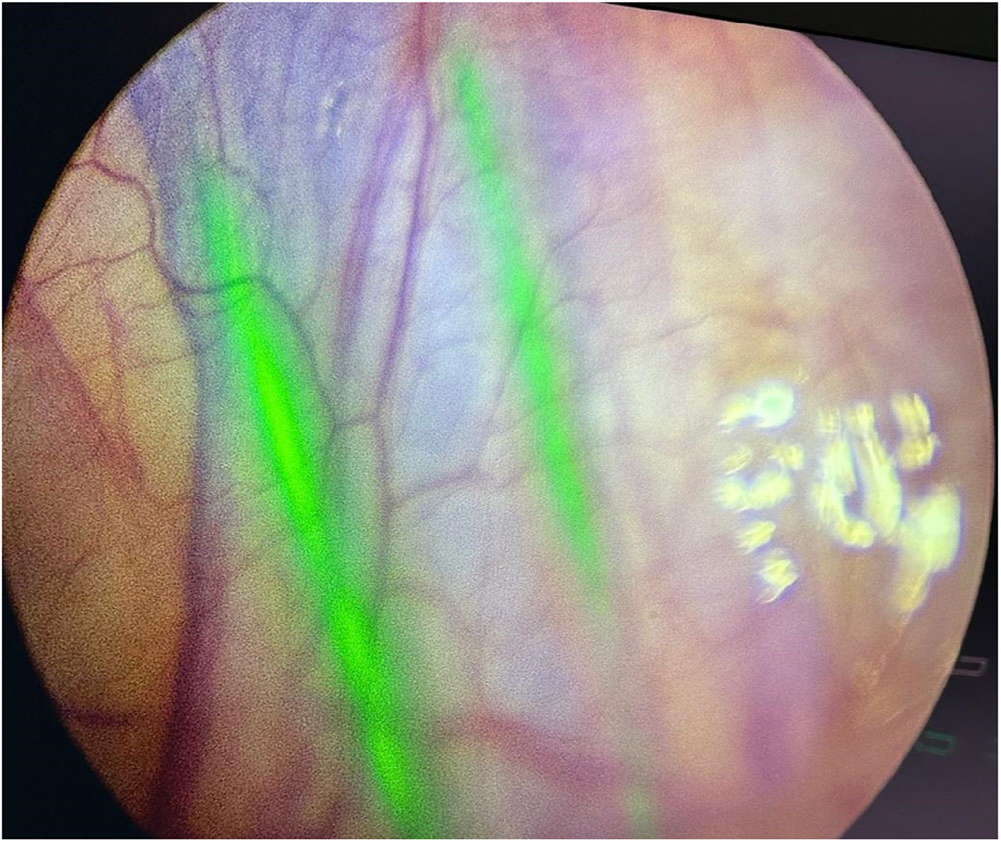
Fluorescence of lymphatic vessels during varicocele surgery.

**Figure 9:**
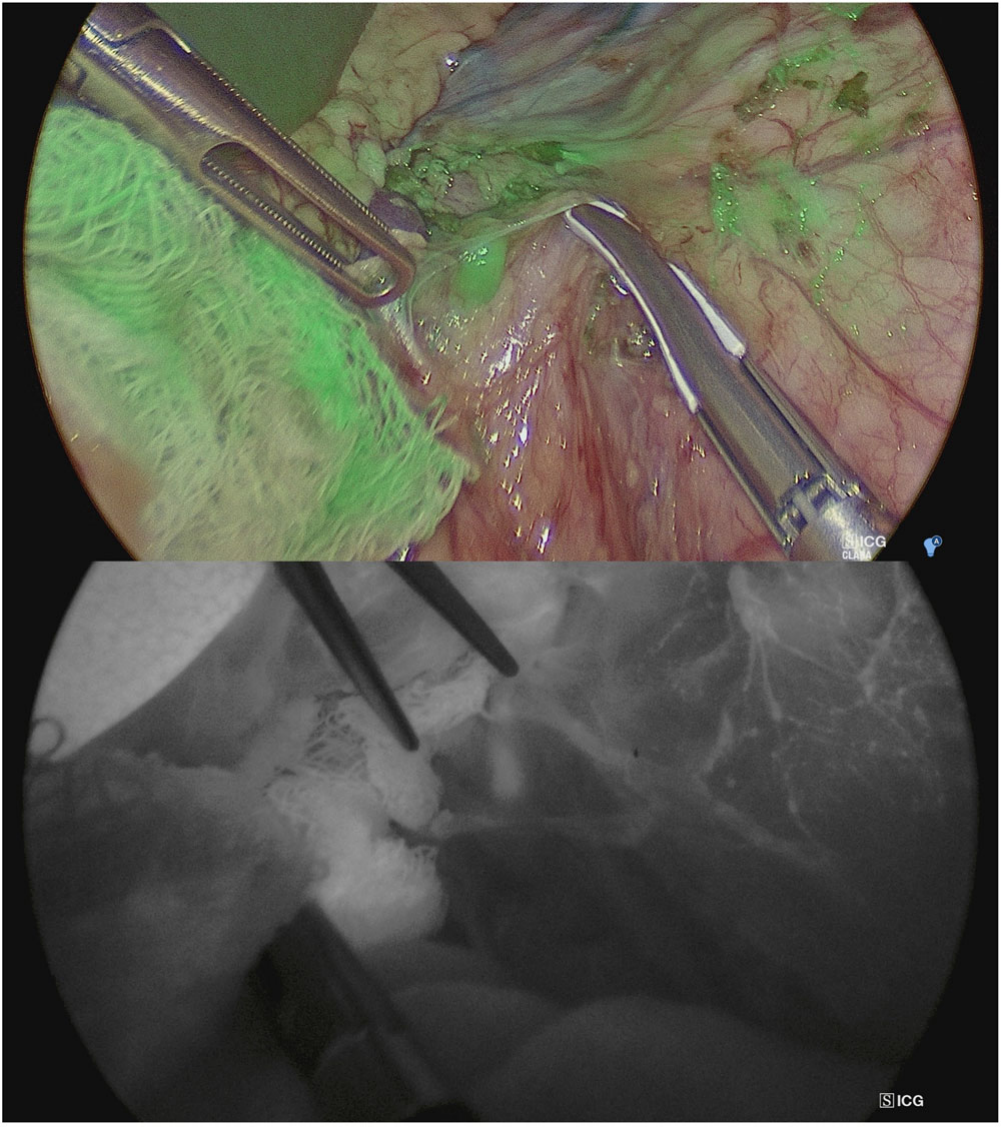
Lymph node fluorescence during resection of a nephroblastoma‐like renal tumor. Images are also captured in monochromatic mode.

**Figure 10:**
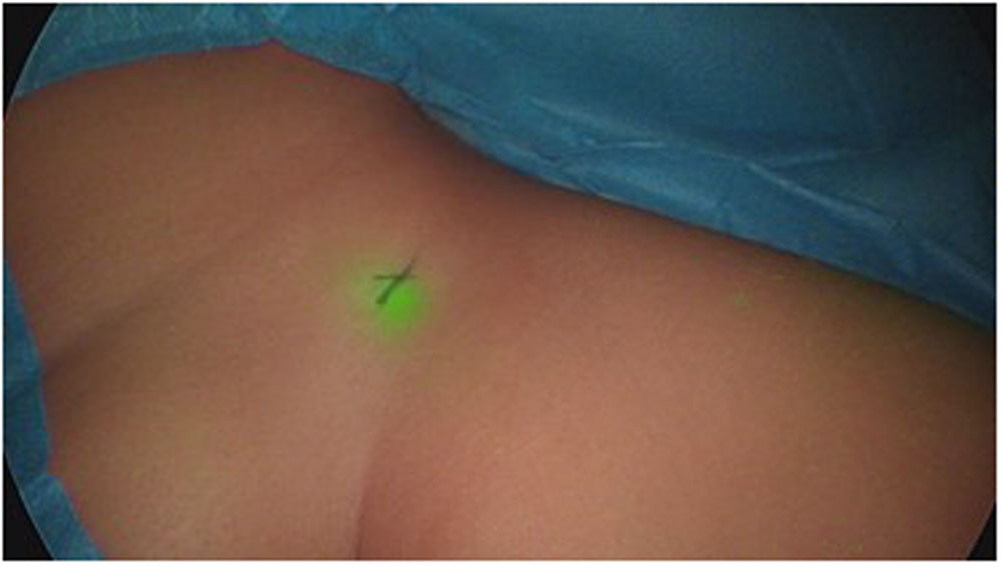
Fluorescence of the sentinel node in the inguinal region in melanoma of the knee.

## 9 Failed Visualization After Indocyanine Green Administration

## 10 Study Limitations

This study has several limitations inherent to its retrospective design. Firstly, the data were collected from medical records, which may be subject to inaccuracies or inconsistencies, potentially impacting the reliability of the findings. Secondly, the lack of randomization and control groups limits the ability to establish causal relationships between the use of ICG and the observed outcomes. Additionally, the study relied on descriptive statistics without comprehensive multivariate analyses to account for confounding variables, which could have influenced the results. Furthermore, the variability in fluorescence imaging equipment and ICG application protocols across centers introduces heterogeneity, making direct comparisons challenging (e.g., instrumentation malfunction, ambient light, distance the SPY‐PHI is held from the target for imaging, intensity settings of the SPY‐PHI etc.). Finally, as a nationwide multi‐center study, differences in expertize, training, and subjective interpretation among surgeons could have impacted the consistency of ICG utilization and outcome assessment.

False positives and false negatives are also limitations of this study. They describe errors in the context of binary classification problems. A false positive is a result where the model incorrectly predicts a positive outcome when the true outcome is negative. A false negative is when the model incorrectly predicts a negative outcome when the true outcome is positive (Table 4).

These limitations underscore the need for prospective, controlled trials to validate the findings and establish standardized protocols for the use of ICG in pediatric surgery.

## 11 Discussion

This nationwide, multicenter cohort study demonstrates the potential benefits and practical applications of ICG fluorescence imaging in pediatric surgery. The study provides compelling evidence of the efficacy of ICG, particularly in enhancing surgical precision and improving postoperative outcomes. These findings are critical given the unique anatomical and physiological challenges of pediatric patients [1, 10]. At the same time, the limitations of ICG use have been highlighted.

ICG fluorescence imaging has proven invaluable in improving intraoperative visualization, which is especially critical in pediatric surgery due to the small size and delicate nature of pediatric anatomical structures. During cholecystectomy, accurate identification of the biliary anatomy using ICG may prevent iatrogenic injury in pediatric surgery [11, 12, 13]. Ciro et al. highlight the ability to overlay ICG‐NIR data onto a standard white light image. This provides surgeons with continuous fluorescence imaging, allowing them to assess the position of critical biliary structures even in the presence of anatomical abnormalities, thus contributing to safer surgery [12]. In gastrointestinal surgery, ICG's ability to assess bowel perfusion ensures viability of the intestine, proper resection margins and anastomotic sites, thereby reducing the risk of anastomotic leakage and other complications [4, 5]. Breuking et al. suggest that ICG may be useful for intraoperative assessment of bowel perfusion, perhaps even more so than conventional clinical assessment [4]. During esophageal reconstuctions using small or large bowel, it may also help in the selection of an intestinal insert and assess its vascularization after transposition to the chest. The surgeon can observe fluorescent confirmation of normal blood supply [14, 15, 16, 17]. The above applications were confirmed in the studied cohort as we assessed ICG perfusion in four anastomoses (two esophageal and two intestinal). In all cases, the perfusion pattern was reported as correct, and no complications occurred (e.g., leakage).

The use of ICG in oncologic surgery has been particularly transformative. The dye's ability to delineate tumor margins and identify sentinel lymph nodes can aid in accurate staging and complete resection of malignant tissue. This precision minimizes the need for reoperations and potentially improves survival outcomes [18, 19, 20]. Koh et al. suggest that in adult patients, sentinel lymph node mapping is an acceptable alternative to routine lymphadenectomy in the surgical staging of early endometrial cancer and is much less invasive than pelvic lymph node dissection [14]. Shen et al. suggest that ICG fluorescence imaging is useful in the resection of HB and may detect small lesions not seen on preoperative imaging [15]. The data of the study showed the high efficacy of ICG in identifying malignant tissues, although some cases of false positives and negatives were noted [21, 22, 23]. Despite various applications the use of ICG also comes with limitations. Majlesara et al. describe that deeper tissues cannot be visualized with ICG due to limited tissue penetration of 5–10 mm. They reported many instances of false positive or negative results [22]. Rodriguez et al. present in their material that of the 11 malignant liver tumors, six were visible on fluorescence imaging. Of the nine benign liver lesions, five were visible. The sensitivity of ICG fluorescence was 45.5%, the specificity 21.2%, the positive predictive value 25% and the negative predictive value 40% [23]. In our material (136 children), there were 9 patients with a false positive result and 5 patients with a false negative result (Table 4). These results underscore the importance of surgeons being skilled in the interpretation of fluorescence patterns to avoid diagnostic inaccuracies.

In urological procedures, such as pyeloplasty and nephrectomy, ICG fluorescence facilitates the identification of ureters and renal vasculature, thereby improving surgical safety and efficacy [8, 24]. Esposito et al. suggest that ICG‐NIRF technology has proven to be safe, easy to use, not time‐consuming, inexpensive, and very effective in improving intraoperative view and surgical skills [8]. In the material we studied, we also confirmed the importance of ICG during surgery for varicocele and removal of the kidney in agenesis.

The effectiveness of ICG utilization improves with the surgeon's experience. However, this often leads to subjective perceptions, and as our own experience suggests it can be a fluid and ambiguous assessment. The application of ICG in oncological surgery, particularly in pediatric oncology, remains largely uncharted. While various devices are available for detecting near‐infrared fluorescence, each has its own proprietary visualization modes and modalities, which are often incomparable. Moreover, there are no extensive studies that clearly define specific doses of ICG or the optimal timing for its administration Despite our 5 years of experience, we still occasionally encounter situations where we question whether the lack of fluorescence is due to the dye's suitability for visualization or if the error lies in our procedure. Therefore, we have included our positive and proven experience in the tables to provide guidance to physicians using ICG.

A significant contribution of this study is the emphasis on standardizing ICG use in pediatric surgery. The detailed protocols for ICG administration, compiled from multiple centers, aim to establish best practices. This standardization is crucial for ensuring consistent outcomes across different surgical teams and settings [25, 26, 27, 28].

Hospitals and surgical centers considering the adoption of this technology must weigh the initial and ongoing costs against the potential improvements in surgical outcomes and operational efficiency. In our opinion, the cost of this method is not neglectable. The availability of specialized fluorescence imaging equipment which is quite expensive may limit its widespread adoption, although currently, the cost of indocyanine green preparation is lower. Centers with limited financial resources that will not fully utilize various ICG applications should consider whether this technique is clinically justifiable in their situation. In addition, regional differences in availability highlight the importance of addressing global disparities in access to advanced medical technologies [29, 30]. Addressing these challenges through continued research and technological advancements is crucial for broader implementation [28, 30, 31, 32].

In our opinion the use of ICG should probably become a standard in certain procedures where its application and usefullness is well proved as eg.: cholecystectomy, bile duct cyst excision, malignant liver tumor resections, esophageal reconstructions with the intestinal insert. In many other indications this method is certainly promising for the future, but, still requires additional investigation.

The findings from this study pave the way for further research into ICG applications in pediatric surgery. Future studies could focus on the long‐term outcomes of ICG‐guided surgeries and explore their potential in other surgical fields. Innovations in fluorescence imaging technology, such as more sensitive and specific dyes or improved imaging systems, could further enhance the efficacy of ICG. Additionally, expanding training programs to include ICG techniques will be essential for integrating this technology into routine pediatric surgical practice [28, 33, 34].

## 12 Conclusion

In conclusion, this Polish nationwide multi‐center cohort study validates the efficacy and safety of ICG fluorescence imaging in pediatric surgery. By enhancing surgical precision and improving postoperative outcomes, ICG represents a significant advancement in pediatric surgical care. The establishment of standardized protocols and the emphasis on specialized training will be key to maximizing the benefits of ICG. As technology and techniques continue to evolve, ICG is poised to play an increasingly important role in improving surgical outcomes for pediatric patients. Surgeons experience remains essential for the correct interpretation of the intraoperative fluorescence.

## Author Contributions

All authors have read and approved the final version of the manuscript had full access to all of the data in this study and take complete responsibility for the integrity of the data and the accuracy of the data analysis.

## Ethics Statement

The study was performed according to the Helsinki Declaration and institutional review board approval was obtained.

## Conflicts of Interest

The authors declare no conflicts of interest.

## Transparency Statement

The lead author Grzegorz Kowalewski affirms that this manuscript is an honest, accurate, and transparent account of the study being reported; that no important aspects of the study have been omitted; and that any discrepancies from the study as planned (and, if relevant, registered) have been explained.

## Data Availability

The data that support the findings of this study are available on request from the corresponding author. The data are not publicly available due to privacy or ethical restrictions.
